# Immigration-related Problems as a Barrier To Meeting the Mental Health Needs of Latinx Youth in Immigrant Families

**DOI:** 10.1007/s10903-025-01784-w

**Published:** 2025-09-24

**Authors:** Yazmin Meza Lazaro, Stephanie H. Yu, Blanche Wright, Laurel Bear, Anna S. Lau

**Affiliations:** 1 University of California, Los Angeles, Los Angeles, USA; 2 University of California, San Francisco, San Francisco, USA; 3 University of Oregon, Eugene, USA; 4 RAND Corporation, Santa Monica, USA; 5 Los Angeles County Office of Education, Downey, USA

**Keywords:** Immigration stress, Latinx youth, Help-seeking, Internalizing symptoms

## Abstract

The increase of anti-immigrant policies and rhetoric creates a heightened sense of fear for Latinx undocumented and mixed status families. Yet, there are numerous barriers that prevent Latinx youth from seeking mental health services (MHS) when in need. This study examined whether immigration-related problems and worry (IPW) during an era of high visibility immigration enforcement moderated the relationship between mental health (MH) need and help-seeking from different sources of support among 4th-12th grade students who self-identified as Latinx first- or second-generation immigrants (*N* = 445). We hypothesized that Latinx youth who experienced IPW would report predominantly relying on peer and non-professional adult support, rather than professional support. Results revealed a significant moderation effect, such that the association between internalizing problems (i.e., MH need) and adult help-seeking was attenuated among Latinx youth who endorsed IPW, compared to those who did not endorse these concerns. These findings suggest that experiencing IPW, stemming from anti-immigrant policies and rhetoric, deter Latinx youth from seeking support from trusting adults (e.g., parents, relatives, teachers), preventing them from receiving their support and getting connected to professional MHS.

Anti-immigrant sentiment spiked with Trump 2016–2020 administration’s rhetoric and policies, impacting youth well-being and heightening fear among undocumented and mixed status families [1, 2]. Immigrants and their children were already less likely than non-immigrants to use MHS [3], and, as such, this study examined whether immigration-related problems or worry (IPW) further undermines youth help-seeking.

From 2016 to 2020, the administration threatened immigration raids, and expanded Immigration and Customs Enforcement (ICE) to ramp up arrests, family separation, and deportation [4, 5]. In California, the state in which this study was conducted, 42% of Latinx individuals knew someone who was deported, and 17% reported seeing ICE in their neighborhood in 2018–2019 [6]. Anti-immigrant policies produced fear, uncertainty, and precarity for undocumented and mixed status families [7]. Many Latinx youth in immigrant families worry about detention, deportation, and separation, causing anxiety and lower sleep quality [8, 9]. The deportation of a loved one is associated with depression, suicidal ideation, and alcohol use among youth [1, 10]. With the second Trump administration executing “Operation At Large” in 2025, an unprecedented crackdown apprehending noncriminal immigrants at work sites, routine immigration office check-ins, and across neighborhoods, it is of renewed importance to understand the impact of this climate on youth mental health.

Anti-immigrant policies and rhetoric produce structural and interpersonal discrimination that impact families [11, 12]. The Public Charge Rule^1^ systemically discriminates against immigrants by deterring them from using available public services. Immigrants without citizenship are deterred from utilizing public services to avoid designation as a ‘public charge’ which could jeopardize later application for Legal Permanent Residency [13]. Although the policy has changed and applies only to certain noncitizen groups, misrepresentations of the rule prevent many from using public benefits, including MHS.

Anti-immigrant policies also foment hostile environments that place immigrant communities in harm’s way [14]. After Trump’s 2016 election, immigrant mothers reported an increase in discrimination [9] and everyday discrimination mediated the link between fear of immigration enforcement and anxiety among Latinx youth [11]. Anti-immigrant policy creates and maintains structural and everyday discrimination that directly and indirectly harm the well-being of immigrant youth.

Despite experiencing distress, Latinx immigrants and their children are less likely than non-immigrants to utilize MHS when in need [3, 15]. This may be due to structural access barriers, including cost, insurance requirements, and shortage of linguistically and culturally responsive services [15, 16]. Moreover, undocumented and mixed status families report fear of deportation as a barrier to utilizing MHS [17, 18]. In 2018–2019 survey, 38% of Latinx immigrants in California reported they were not protected from immigration officials at clinics [12]. Another study of Latinx caregivers found that 23% feared being deported if they sought support for their children [18].

Given the increased risk of MH concerns in anti-immigrant context and linked barriers to MHS, it is crucial to understand where immigrant youth seek support when in need. Latinx adolescents seek support most often from friends, followed by trusted adults and caregivers, and least often from MHS [19, 20]. Immigrant communities often uphold interdependent cultural values and those who endorse *familismo* are less likely to seek MHS compared to informal support or religious guidance [21, 22]. Family obligation concerns often prevent immigrant youth from seeking support when in need [23]. Latinx youth facing IPW in their families may be unlikely to seek support as they may avoid sharing their problems with their caregivers due to concerns about adding burden [24]. We sought to understand how IPW might shape or suppress help-seeking among distressed Latinx youth.

The current study examined: (1) where Latinx youth in immigrant families sought support depending on whether they experienced past-year IPW, and (2) whether IPW weakened the relationship between MH need and help-seeking. We hypothesized that Latinx youth would predominantly rely on peer and non-professional adult support rather than professional support if they had experienced IPW. Additionally, we hypothesized the relationship between internalizing symptoms and help-seeking would be attenuated when youth endorsed IPW.

## Method

### Data Source

Data were collected by a school district in Southern California serving low-income and immigrant families, with about 42% of students identifying as Latinx. All schools in the district were Title I eligible and 70–81% of students received free/reduced cost lunch. The district was supported by grants, partnerships, and their Local Control and Accountability Plan to develop, implement, and evaluate a comprehensive school-based MHS system [25].

### Procedures

The district surveyed 2,681 students within a stratified random sample of 4th−12th grade classrooms across 18 schools in 2018–2019 [26]. The University of California, Los Angeles Institutional Review Board determined the study was exempt and waived requirements for consent since it was a secondary analysis of anonymous data collected for a school district’s program evaluation.

### Participants

Students who self-identified as Latinx who were either immigrants themselves or had at least one parent/guardian who is an immigrant (*N* = 445) were included in the analyses. Most were second-generation immigrant youth (*n* = 414; 93%), about half were girls (*n* = 230; 51.7%) and high school students (*n* = 248; 55.8%). See Table 1.

**Table 1:** Descriptive Information of the Sample (*N* = 445)

Variable	*n* (%)
Grade Level	
Grades 4 through 8	197 (44.2)
Grades 9 through 12	248 (55.8)
Gender	
Girls	230 (51.7)
Boys	203 (45.6)
Prefer not to say	12 (2.7)
Student Immigrant Generation	
First generation	31 (7.0)
Second generation	414 (93.0)

## Measures

### Immigration-Related Problems and Worry

We created a composite to index IPW. For immigration problems, we used one item: “Did someone close to you have problems related to their immigration status or being able to stay in the country.” (0 = *no*, 1 = *yes*). Immigration-related worry was assessed with an item from the Bicultural Stress Scale [27] rated from 0 (*never happened*) and 1 (not at all stressful) to 5 (*very stressful*): “I have worried about family members or friends having problems with immigration.” Given the correlation between immigration problems and worry (*r* = 0.413, *p* < 0.01), we created a single composite binary variable. Participants who endorsed immigration problems or at least some immigration-related worry (i.e., indicated their worry about their family members or friends having problems with immigration were either a little, quite a bit, or very stressful) during the last year were coded as endorsing past-year IPW (0 = *no*; 1 = *yes*). We relied on indirect single-item measures, rather than asking directly about youth and family documentation status to avoid placing participants at risk [28].

### Internalizing Symptoms

We assessed internalizing symptoms using the Emotional Problems subscale from the Strengths and Difficulties Questionnaire (SDQ) [29]. Participants rated 5 items on a scale from 0 (*not true*) to 2 (*certainly true*) (e.g., “I worry a lot,”) Internal consistency was adequate, Cronbach’s alpha was 0.71.

### Help-Seeking

Participants completed a modified version of the General Help-Seeking Questionnaire [30] previously used with youth in a study by Guo and colleagues [23]. These modifications included changing the scale from a 1 to 7 Likert scale to a dichotomous yes or no scale, making slight wording changes to a few items (e.g., intimate partner was changed to partner (boy/girlfriend)), and adding youth worker as a possible source of support. Each of these modifications were made to tailor the measure for youth. We asked participants whether they sought help from 11 sources of support in the past year (0 = *no*, 1 = *yes*). Based on prior factor analysis in the same school district context [26], items were grouped the sources into three categories: professional support (i.e., MH professional, academic counselor, doctor, or help line), non-professional adult support (i.e., parent, family member (non-parent), teacher, pastor/priest, youth worker), and peer support (i.e., romantic partner or friend). We created a four-level multinomial outcome variable based on the highest intensity of support sought in the following order: (1) no support sought, (2) only sought peer support, (3) sought support from an adult but not professional help, regardless of whether peer support was sought, and (4) sought professional help, regardless of whether adult or peer support was sought [23].

## Results

As a preliminary analysis, the variability in immigration-related problems and worry, was explored. For immigration-related problems, 78 participants endorsed a problem (17.5%) and 367 endorsed no problem (82.5%). For immigration-related worry, 147 participants endorsed worry (33%) and 298 endorsed no worry (67%). However, 89 of the 367 that reported no immigration-related problems endorsed immigration-related worry. As such, 167 participants (37.4%) endorsed IPW and 278 participants (62.6%) did not endorse IPW. We found no significant differences in support sources between youth who did and did not endorse IPW, χ^2^(3) = 0.91, *p* = 0.823. See Table 2.

**Table 2:** Crosstabs of Help-Seeking by IPW (*N* = 445)

Type of Help-Seeking	No Past-Year IPW *n* (%)	Past-Year IPW *n* (%)
No support	86 (31%)	45 (27%)
Peer support	30 (11%)	18 (11%)
Non-professional Adult support	105 (38%)	69 (41%)
Professional support	57 (20%)	35 (21%)

In the first step of the multinomial logistic regression, we entered the control variables (i.e., gender, age, and youth’s immigrant generation), internalizing symptoms, and IPW into the model, with help-seeking as the dependent variable, χ^2^(18) = 67.5, *p* < 0.001, AIC = 1,125.49. There was main effect of symptoms on all levels of help-seeking, such that a one standard deviation increase in symptoms was associated with a 47% increase in the likelihood of seeking peer support (SE = 0.15, *p* < 0.001, 95% CI=[1.20, 1.79), a 42% increase in the likelihood of seeking non-professional adult support (SE = 0.11, *p* < 0.001, 95% CI=[1.21, 1.66]), and a 51% increase in the likelihood of seeking professional support (SE = 0.13, *p* < 0.001, 95% CI=[1.26, 1.79]) compared to seeking no support.

In the next step, we added the interaction term between internalizing symptoms and IPW, χ^2^(21) = 76.15, *p* < 0.001, AIC = 1,122.19. The likelihood ratio test indicated that the interaction between internalizing symptoms and IPW significantly increased model fit, χ^2^ (3) = 8.65, *p* = 0.0343. IPW moderated the association between internalizing symptoms and seeking support from adults compared to seeking no support, by decreasing the likelihood by 77% (SE = 0.09, *p* = 0.032, 95% CI=[0.60, 0.98]; See Table 3).

**Table 3:** Hierarchical Multinomial Logistic Regression Models for Help-Seeking Sources

	No help vs. peer				No help vs. adult				No help vs. professional			
			95% CI OR				95% CI OR				95% CI OR	
Highest intensity of support sought	*RRR*	SE	LL	UL	*RRR*	SE	LL	UL	*RRR*	SE	LL	UL
**Model 1**												
Grade	1.05	0.07	0.91	1.2	0.94	0.05	0.85	1.03	0.89	0.05	0.79	0.99
Gender												
Boys (reference)												
Girls	0.97	0.36	0.46	2.01	1.18	0.31	0.71	1.96	1.34	0.42	0.72	2.48
Prefer Not to Say	1.30	1.57	0.12	13.90	0.51	0.49	0.08	3.30	5.46	4.33	1.15	25.85
Youth Immigrant Generation	0.34	0.23	0.09	1.29	0.47	0.26	0.16	1.38	0.31	0.19	0.09	1.05
IS	1.47***	0.15	1.20	1.79	1.42***	0.11	1.21	1.66	1.51***	0.13	1.26	1.79
IPW	0.75	0.28	0.36	1.56	0.92	0.25	0.54	1.55	0.60	0.20	0.31	1.17
**Model 2**												
ISxIPW	0.83	0.14	0.60	1.14	0.77*	0.09	0.60	0.98	1.03	0.15	0.78	1.36

IS = Internalizing Symptoms, IPW = Immigration-related Problems and Worry, RRR = Relative Risk Ratio, SE = Standard Error, LL = Lower Limit, UL = Upper Limit, *=< 0.05, ***=< 0.001

**Fig. 1:**
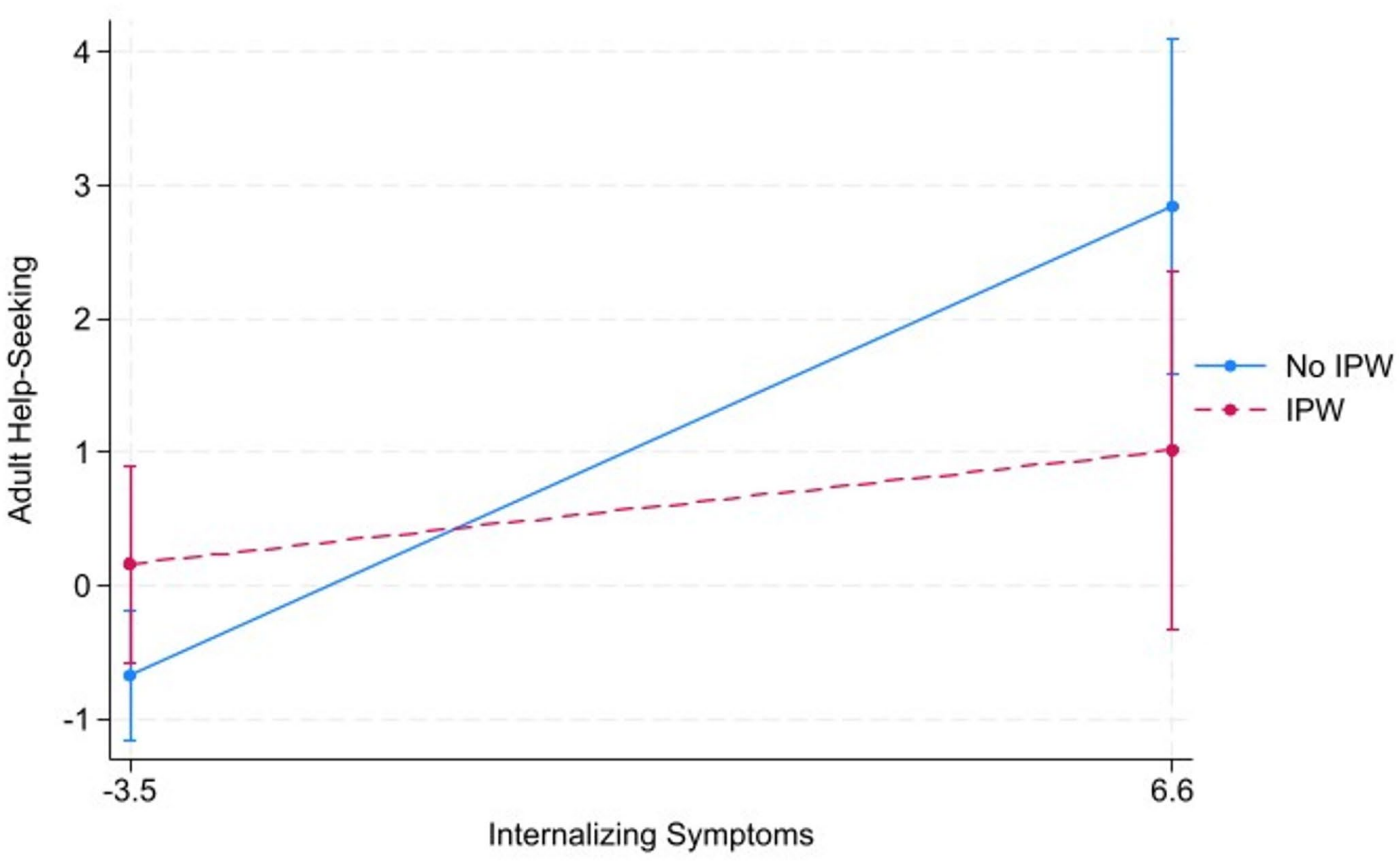
Relationship Between Internalizing Symptoms and Adult Help Seeking Moderated by IPW

Simple slope analysis found that the likelihood of seeking non-professional adult support increased by 42% with a one unit increase in internalizing symptoms among youth not reporting IPW (SE = 0.11, *p* < 0.001, 95% CI=[1.21, 1.66]). Among youth who endorsed IPW, there was no association between seeking non-professional adult support and internalizing symptoms. See Fig. 1 (created using IBM SPSS).

## Discussion

We examined whether IPW prevented Latinx youth in immigrant families from seeking help under distress. Latinx youth in our sample most frequently relied on non-professional adult support as the highest intensity of support sought, regardless of whether they reported past-year IPW. However, the chance that a participant sought out non-professional adult support only increased as a function of MH need for youth who did not report past-year IPW. Thus, IPW functioned as a barrier to seeking support from trusted adults for Latinx youth in need.

Although we predicted Latinx youth who experienced IPW would be more likely rely on peer and non-professional adult support than professional MHS, we ultimately found no differences in patterns of help-seeking compared to Latinx youth who did not report IPW. Across groups, seeking support from adults as the ‘highest intensity’ was the most common, followed by seeking support from no one, professional support as the ‘highest intensity’, and finally peer support alone. This was consistent with findings that youth in immigrant communities are less likely to seek MHS compared to informal adult support. This may be jointly explained by structural access barriers [15, 16], and values that prioritize seeking support within the family or community [21, 22].

Our hypothesis that IPW would attenuate the link between MH need and help-seeking behaviors was supported. Seeking non-professional adult support only increased with greater symptoms among youth who did not endorse past-year IPW. Having loved ones who are experiencing IPW can reduce the chances of youth seeking support when they are in need [23]. Latinx youth who are aware of or worried about immigration problems faced by their loved ones may not want to further burden the adults in their life. Even with non-familial adults (e.g., teachers and religious leaders), youth may be worried that their family will learn of their problems, ultimately adding to their stress or attracting attention to the family. Asking for support from trusted adults may feel risky for youth experiencing IPW during times of rising anti-immigrant rhetoric [4, 7].

The finding that IPW suppressed the typical link between MH need and seeking support from caring adults has critical implications. The stress of witnessing IPW in their circle prevents Latinx youth from communicating their emotional stress, and as a result, can rob them of protective support from their trusted adults. Familial and community support are critical for the well-being of Latinx youth [17, 24] and caring adults play a key role in connecting youth to MHS [21]. Contrary to predictions, IPW did not moderate the link between need and seeking professional care. This may be related to prior findings that immigrant communities are less likely to seek professional services overall [3, 13, 15]. Alternatively, since the current study’s school district context included access to a multi-tier system of mental health support, this may have removed many access barriers for students in immigrant families including the promotion of services and access to community members who were educated on how to refer students and request care [25]. With accessible confidential services available, IPW may not have deterred their use.

### Limitations

These findings should be interpreted within limitations. First, our IPW measure was a practical composite of two items that could be answered by youth while protecting their privacy within a school district’s climate assessment. However, this minimal approach did not allow us to examine who in the student’s network was experiencing immigration status precarity, including whether or not the students themselves were undocumented. The use of the composite also did not allow us to determine whether knowledge of immigration status problems versus worries about potential problems are driving the observed effects. Future studies should use a measure that disentangles the individual effects of problems and worries. It is also a priority to develop brief pragmatic measures of immigration related stress that are scalable in systems serving youth. Second, although participants identified as Latinx, the experiences of Latinx communities are heterogeneous and we did not assess country of origin or other aspects of their history and lived experience in the U.S [31]. Third, the study was conducted in lower-income neighborhoods in a region close to the U.S.-Mexico border and may not generalize to other contexts. Lastly, the study relies on self-reported, cross-sectional data.

### Implications and Future Directions

Our study expanded the literature by exploring how IPW impacts help-seeking among Latinx youth in immigrant families. The sociopolitical context of 2018–2019 may reflect how anti-immigrant sociopolitical structures [8, 9] shape IPW and its cascading effects on Latinx youth. We report a novel finding that IPW suppresses the likelihood that Latinx youth will seek support from trusted adults when in need. The results call for school personnel and trusted adults in the lives of Latinx youth to act to acknowledge the strain and fear of immigration enforcement actions and the general precarity of having family or loved ones who are undocumented. Teachers and other school leaders, for example, can play a role in organizing and hosting “Know Your Rights” campaigns, ensuring schools are safe spaces for undocumented youth and their families, and refusing cooperation with ICE. Trusted adults within and outside the education system can also provide direct support and connect youth and families to services they need. Action at the structural level is also needed to prevent the erosion of this crucial support.

## Data Availability

No datasets were generated or analysed during the current study.

## Footnotes

1 The 1999 Interim Field Guidance defined public charge as a non-citizen who had become or was likely to become primarily dependent on the government, demonstrated by using public cash assistance for income maintenance or institutionalization for long-term care at the government’s expense. This was in place until the 2019 Public Charge Final Rule was implemented in February 2020 included non-cash benefits such as Medicaid, Supplemental Nutrition Assistance Program (SNAP), and Public Housing. This rule was in place until March 2021.

## References

[1] Giano Z, Anderson M, Shreffler KM, Cox RB Jr, Merten MJ, Gallus KL. Immigration-related arrest, parental documentation status, and depressive symptoms among early adolescent Latinos. Cultur Divers Ethnic Minor Psychol. 2020;26(3):318. 10.1037/cdp0000299. 31368725 10.1037/cdp0000299 PMC6994349

[2] Wright B, Chen B, Kodish T, Meza Lazaro Y, Lau AS. Immigration stress and internalizing symptoms among Latinx and Asian American students: the roles of school climate and community violence. J School Psychol. 2024;104:101286. 10.1016/j.jsp.2024.101286. 10.1016/j.jsp.2024.101286 38871411

[3] Finno-Velasquez M, Cardoso JB, Dettlaff AJ, Hurlburt MS. Effects of parent immigration status on mental health service use among Latino children referred to child welfare. Psychiatr Serv. 2016;67(2):192–8. 10.1176/appi.ps.201400444. 26467910 10.1176/appi.ps.201400444

[4] Ayón C. Talking to Latino children about race, inequality, and discrimination: raising families in an anti-immigrant political environment. J Soc Social Work Res. 2016;7(3):449–77. 10.1086/686929.

[5] Ryo E. How ICE enforcement has changed under the Trump administration. *The Conversation*. 2019. https://theconversation.com/how-ice-enforcement-has-changed-under-the-trump-administration-120322. Accessed 15 June 2024.

[6] Young M-E, Tafolla S. Latinx and Asian immigrants across California regions have different experiences with law and immigration enforcement. *UCLA Center for Health Policy Research*. 2021. https://healthpolicy.ucla.edu/publications/search/pages/detail.aspx?PubID=2225. Accessed 15 June 2024.

[7] Barajas-Gonzalez RG, Ayón C, Brabeck K, Rojas-Flores L, Valdez CR. An ecological expansion of the adverse childhood experiences (ACEs) framework to include threat and deprivation associated with US immigration policies and enforcement practices: an examination of the Latinx immigrant experience. Soc Sci Med. 2021;282:114126. 10.1016/j.socscimed.2021.114126. 34146987 10.1016/j.socscimed.2021.114126 PMC10409596

[8] Eskenazi B, Fahey CA, Kogut K, Gunier R, Torres J, Gonzales NA, Deardorff J. Association of perceived immigration policy vulnerability with mental and physical health among US-born Latino adolescents in California. JAMA Pediatr. 2019;173(8):744–53. 10.1001/jamapediatrics.2019.1475. 31233132 10.1001/jamapediatrics.2019.1475 PMC6593622

[9] Singer MA, Velez MG, Rhodes SD, Linton JM. Discrimination against mixed-status families and its health impact on Latino children. J Appl Res Child. 2018;10(1). 10.58464/2155-5834.1364. PMC6746556 31528499

[10] Roche KM, White RM, Lambert SF, Schulenberg J, Calzada EJ, Kuperminc GP, Little TD. Association of family member detention or deportation with Latino or Latina adolescents’ later risks of suicidal ideation, alcohol use, and externalizing problems. JAMA Pediatr. 2020;174(5):478–86. 10.1001/jamapediatrics.2020.0014. 32176245 10.1001/jamapediatrics.2020.0014 PMC7076534

[11] Cardoso JB, Brabeck K, Capps R, Chen T, Giraldo-Santiago N, Huertas A, Mayorga NA. Immigration enforcement fear and anxiety in Latinx high school students: the indirect effect of perceived discrimination. J Adolesc Health. 2021;68(5):961–8. 10.1016/j.jadohealth.2020.08.019. 33139180 10.1016/j.jadohealth.2020.08.019

[12] Pourat N, Young M-E, Morales B, Chen L. Latinx and Asian immigrants have negative perceptions of the immigrant experience in California. *UCLA Center for Health Policy Research*. 2021. https://healthpolicy.ucla.edu/publications/search/pages/detail.aspx?PubID=2223. Accessed 15 June 2024.

[13] Awaad R, Dailami M, Noureddine N. US policy of public charge inadmissibility and refugee suicides. Lancet Psychiatry. 2020. 10.1016/s2215-0366(20)30037-7. 32087811 10.1016/S2215-0366(20)30037-7

[14] Almeida J, Biello KB, Pedraza F, Wintner S, Viruell-Fuentes E. The association between anti-immigrant policies and perceived discrimination among Latinos in the US: a multilevel analysis. SSM Popul Health. 2016;2:897–903. 10.1016/j.ssmph.2016.11.003. 29349196 10.1016/j.ssmph.2016.11.003 PMC5757908

[15] Derr AS. Mental health service use among immigrants in the United States: a systematic review. Psychiatr Serv. 2016;67(3):265–74. 10.1176/appi.ps.201500004. 26695493 10.1176/appi.ps.201500004 PMC5122453

[16] Alegría M, Mulvaney-Day N, Woo M, Torres M, Gao S, Oddo V. Correlates of past-year mental health service use among latinos: results from the National Latino and Asian American study. Am J Public Health. 2007;97(1):76–83. 10.2105/ajph.2006.087197. 17138911 10.2105/AJPH.2006.087197 PMC1716237

[17] Benavides Q, Doshi M, Valentín-Cortés M, Militzer M, Quiñones S, Kraut R, et al. Immigration law enforcement, social support, and health for Latino immigrant families in southeastern Michigan. Soc Sci Med. 2021;280:114027. 10.1016/j.socscimed.2021.114027. 34029864 10.1016/j.socscimed.2021.114027 PMC8525509

[18] Vázquez AL, Culianos D, Flores CMN, Alvarez MDLC, Barrett TS, Domenech Rodríguez MM. Psychometric evaluation of a barriers to mental health treatment questionnaire for Latina/o/x caregivers of children and adolescents. Child Youth Care Forum. 2022;51(4):847–64. 10.1007/s10566-021-09656-8. 34642563 10.1007/s10566-021-09656-8 PMC8494628

[19] De Luca SM, Lim J, Yueqi Y. Young adolescents’ help seeking behaviors and attitudes: an examination of an underserved community. Child Adolesc Soc Work J. 2019;36(6):599–607. 10.1007/s10560-019-00604-z. 10.1007/s10560-019-00604-z PMC10817752 38284001

[20] Sabina C, Cuevas CA, Rodriguez RM. Who to turn to? Help-seeking in response to teen dating violence among Latinos. Psychol Violence. 2014;4(3):348. 10.1037/a0035037.

[21] Kuo BC, Roldan-Bau A, Lowinger R. Psychological help-seeking among Latin American immigrants in Canada: testing a culturally-expanded model of the theory of reasoned action using path analysis. Int J Adv Couns. 2015;37(2):179–97. 10.1007/s10447-015-9236-5.

[22] Villatoro AP, Morales ES, Mays VM. Family culture in mental health help-seeking and utilization in a nationally representative sample of Latinos in the United States: the NLAAS. Am J Orthopsychiatry. 2014;84(4):353. 10.1037/h0099844. 24999521 10.1037/h0099844 PMC4194077

[23] Guo S, Nguyen HT, Weiss B, Ngo V, Lau AS. The link between mental health need and help-seeking behavior among adolescents: moderating role of ethnicity and cultural values. J Couns Psychol. 2015;62:682–93. 10.1037/cou0000094. 26376178 10.1037/cou0000094 PMC4605858

[24] Taylor SE, Sherman DK, Kim HS, Jarcho J, Takagi K, Dunagan MS. Culture and social support: who seeks it and why? J Pers Soc Psychol. 2004;87(3):354. 10.1037/0022-3514.87.3.354. 15382985 10.1037/0022-3514.87.3.354

[25] Bear L, Finer R, Guo S, Lau AS. Building the gateway to success: an appraisal of progress in reaching underserved families and reducing racial disparities in school-based mental health. Psychol Serv. 2014;11(4):388. 10.1037/a0037969. 25383994 10.1037/a0037969

[26] Guo S, Kataoka SH, Bear L, Lau AS. Differences in school-based referrals for mental health care: understanding racial/ethnic disparities between Asian American and Latino youth. School Ment Health. 2014;6(1):27–39. 10.1007/s12310-013-9108-2.

[27] Romero AJ, Roberts RE. Stress within a bicultural context for adolescents of Mexican descent. Cultur Divers Ethnic Minor Psychol. 2003;9(2):171. 10.1037/1099-9809.9.2.171. 12760328 10.1037/1099-9809.9.2.171

[28] Allen MS, Iliescu D, Greiff S. Single item measures in psychological science: a call to action. Eur J Psychol Assess. 2022;38(1):1–5. 10.1027/1015-5759/a000699.

[29] Goodman R, Ford T, Simmons H, Gatward R, Meltzer H. Using the strengths and difficulties questionnaire (SDQ) to screen for child psychiatric disorders in a community sample. Br J Psychiatry. 2000;177(6):534–9. 10.1192/bjp.177.6.534. 11102329 10.1192/bjp.177.6.534

[30] Wilson CJ, Deane FP, Ciarrochi JV, Rickwood D. Measuring help seeking intentions: properties of the general help seeking questionnaire. Can J Couns. 2005;39(1):15–28. 10.1037/t42876-000.

[31] Isasi CR, Rastogi D, Molina K. Health issues in Hispanic/Latino youth. J Latina-o Psychol. 2016;4(2):67. 10.1037/lat0000054. 10.1037/lat0000054 PMC4915390 27347457

